# Comprehensive analysis of m6A related gene mutation characteristics and prognosis in colorectal cancer

**DOI:** 10.1186/s12920-023-01509-8

**Published:** 2023-05-16

**Authors:** Tao Jiang, Linshuai Xing, Lipeng Zhao, Ziqi Ye, Dong Yu, Shengtao Lin

**Affiliations:** 1 Department of Anal-Colorectal Surgery, General Hospital of Ningxia Medical University, 804 Shengli Road, Yinchuan, 750004 China; 2 School of Clinical Medicine, Ningxia Medical University, 1160 Shengli Road, Yinchuan, 750004 China; 3 Shengli Clinical Medical College of Fujian Medical University, Fuzhou, 350001 China; 4 Department of Surgical Oncology, Fujian Provincial Hospital, Fuzhou, 350001 China

**Keywords:** Colorectal cancer, Immune, m6A, Prognosis

## Abstract

**Background:**

Colorectal cancer is considered as the second most common cancer worldwide. Studies have shown that m6A RNA methylation abnormalities play an important role in the pathogenesis of many human diseases, including cancer. The current study was designed to characterize the mutation of m6A related genes and explore their prognostic role in colorectal cancer.

**Methods:**

RNA-seq data and somatic mutation data of TCGA-COAD and TCGA-READ were downloaded from UCSC xena for comprehensive analysis. M6A related genes were selected from previous literatures, including "Writer" protein (METTL3, METTL5, METTL14, METTL16, ZC3H13, RBM15, WTAP, KIAA1429), "Reader" protein YTHDF1, YTHDF2, YTHDF3, YTHDC1, YTHDC2, HNRNPC, IGF2BP1, IGF2BP2, IGF2BP3), and "Eraser" protein (FTO, ALKBH5). Kaplan–Meier diagrams were used to explore the correlation between m6A-related genes and colorectal cancer prognosis. The correlations between m6A-related genes and clinical parameters and immune-related indicators were explored by Spearman correlation analysis. And finally, the expression patterns of five key genes (RBMX, FMR1, IGF2BP1, LRPPRC and YTHDC2) were detected by qPCR in CRC specimens.

**Results:**

In CRC, the expressions of m6A-related genes were significantly different between CRC and normal control except METTL14, YTHDF2, YTHDF3. Some of CRC patients (178 in 536) have a m6A-related genes mutation. ZC3H13 has highest mutation frequency of all m6A-related genes. M6A-related genes mainly enrich in regulation of mRNA metabolic process pathway. Patients with high expressions of FMR1, LRPPRC, METTL14, RBMX, YTHDC2, YTHDF2, YTHDF3 have poor prognosis in CRC. There was a significant correlation between the FMR1, LRPPRC, RBMX, YTHDC2, IGF2BP1 expression and the clinical characteristics of CRC. In addition, these genes are significantly associated with immune-related indicators. According to the expression patterns of FMR1, LRPPRC, RBMX, YTHDC2, and IGF2BP1, patients with CRC were clustered into two groups, and their survival was significantly different. By evaluating the tumor microenvironment in two clusters using ssGSEA, expressions of immune checkpoints and GSVA enrichment analysis, we observed that the immune and stem cell index of two cluster were much different. The qPCR results showed that RBMX expression was markedly elevated in cancerous tissues than in the normal colonic tissues.

**Conclusion:**

Our study identified novel prognostic markers associated with immune of CRC cancer patients. Moreover, the potential mechanisms of prognostic markers in regulating the etiology of CRC cancer were investigated. These findings enrich our understanding of the relationships between m6a related genes and CRC, and may provide novel ideas in the therapy of CRC patients.

**Supplementary Information:**

The online version contains supplementary material available at 10.1186/s12920-023-01509-8.

## Background

Colorectal cancer (CRC) is one of the most frequent causes of morbidity and mortality in China [1]. According to the latest data released by the National Cancer Center, CRC is the fourth leading cause of cancer-related death in China, where there are approximately 390,000 new cases and over 196.000 deaths each year [2, 3]. Antineoplastic protocols for CRC have already made the tangible progress in recent years, including Muti-disciplinary treatment, radical operation, neoadjuvant chemotherapy, systematic chemotherapy, radiation treatment, targeted therapy and immunotherapy [4, 5]. The molecular pathogenesis of CRC is heterogeneous [6]. Conventionally, the molecular subtypes of colorectal cancer were simply classified into two categories: microsatellite stable (MSS) or microsatellite instability-Low (MSI-L) and microsatellite instability-high (MSI-H), which characterized by chromosomal changes and DNA mismatch repair deficiency, respectively. Molecular pathogenesis associated with multidisciplinary therapy response and prognosis. Pembrolizumab, a monoclonal antibody for programmed death 1 (PD-1) blockade has clinical benefit in microsatellite-instability-high (MSI-H) tumors after previous therapy, whereas it has no effect in MSI-L or MSS subtypes [7]. Therefore, it is necessary to identify sensitive biomarkers for ascertaining prognosis, monitoring of recurrence and improve personalized therapy managements.

N6-methyladenosine (m6A) methylation, the most common RNA modifications were widely exist in eukaryotic cells. The m6A modification, are key regulatory mechanism in posttranscriptional gene expression control, ranging from RNA stability, cleavage, transport, and RNA translation to decay [8, 9]. It plays important role in biological processes, which affect natural function and metabolism activity, also some kinds of diseases. Several findings suggested that m6A modification play the distinct roles in malignant biological properties, such as stem cell differentiation, metastasis and tumor immune escape [10]. Recently, solid evidence provides the linkage between abnormal regulation of m6A and various kinds of tumor type, including CRC [11–14]. It was reported that m6A methyltransferase METTL14 suppress the malignant biological behavior of gastric cancer by regulating PI3K/AKT/mTOR signaling pathway [15]. As presented in Tsuchiya K’s research, m6A RNA methylation regulators, including YTHDF1 and YTHDF2 are associated with better patient survival and therapeutic targets related to the tumor-immune microenvironment in non-small cell lung cancer [16]. Despite the accumulation of research papers on m6A regulation and cancer, more studies of m6A regulators in CRC are needed to strengthen scientific evidences and support firm conclusions. Therefore, with the applications of bioinformatics, the investigation of RNA-Seq and The Cancer Genome Atlas (TCGA) dataset focuses on the potential biomarkers in the survival outcomes of CRC.

In this study, based on RNA-Seq data and TCGA dataset, the prognostic value of m6A related genes could be acquired by bioinformatics and statistical analysis. Then, five m6A regulators significantly correlated with prognosis were filtrated and employed to construct the m6A related prognostic signature. In addition, the correlations between m6A-related genes and clinical parameters and immune-related indicators were explored to further demonstrate the underlying mechanisms for colorectal cancer.

## Methods

### Data source

Gene expression profile and clinical information of 536 CRC sample were downloaded from TCGA database (http://portal.gdc.cancer.gov/) Somatic cell mutation data is derived from the cBioPortal database (https://www.cbioportal.org/). M6A Regulators include 9 writers (METTL3, METTL14, METTL16, RBM15, RBM15B, WTAP, KIAA1429, CBLL1, ZC3H13), two Erasers (ALKBH5, FTO), and 14 Readers (YTHDC1, YTHDC2, YTHDF1, YTHDF2, YTHDF3, IGF2BP1, IGF2BP2, IGF2BP3, HNRNPA2B1, HNRNPC, RBMX, FMR1, LRPPRC, ELAVL1).

### Correlation study with immune-related indicators

Using the ssGSEA database, we detected the correlation between the expressions of m6A regulators and infiltration levels of immune cells (Cancer associated fibroblast, Myeloid dendritic cell, CD4 T cell, Neutrophil, T cell regulatory (Tregs), CD8 T cell, Macrophage) in CRC. Correlations between the expressions of m6A regulators and immuno regulators (immunosuppressant, immunostimulator, and MHC molecules) were calculated using TISIDB database (TISIDB, an integrated repository portal for tumor-immune system interactions. http://cis.hku.hk/TISIDB/).

### Genetic alteration of m6A regulators in CRC

CBioPortal is an open-access website that explores, visualizes, and analyzes multidimensional cancer genomics data, which was used to analyze the genetic alterations of m6A regulators in CRC.

### Survival analysis

The correlation between m6A regulators aberrations and survival time in human CRC was determined using Kaplan–Meier diagrams. The correlation between overalls survival time and m6A regulators expression are evaluated by Kaplan–Meier diagrams.

### Stem cell index analysis

The dryness index was derived using OCLR (one-class logistic regression) algorithm training and implemented by R Synapser package. Then the transcriptome expression corresponding to the dryness index calculated based on OCLR was applied to the CRC data set to calculate the mRNAsi of each sample. Each dryness index (SI) ranged from low (0) to high (1), and the wilcoxon test was used to test significance between different clusters.

### Patients and specimens for clinical verification

Cancerous tissues and adjacent normal tissues were gathered from 60 CRC patients who received curative surgery from 2018 to 2019 at General Hospital of Ningxia Medical University. All the cases included 34 male and 26 female, which consist of 17 stage II and 43 stage III patients according to the National Comprehensive Cancer Network practice guidelines. No patients had received chemotherapy or radiotherapy before surgery. All frozen tissues were subjected to mRNA extraction for reverse transcription quantitative real-time PCR (RT-qPCR).

### RNA extraction and RT-qPCR

Total RNA was isolated using The TRIzol™ Plus RNA Purification Kit (Thermo Fisher Scientific, Germany), followed by cDNA synthesis for qPCR. RT-qPCR was performed using LightCycler® 480 SYBR Green I Master (Roche, USA) according to the manufacturer’s protocol. The relative RNA amount was calculated using 2−△△Ct comparative method with the normalization to actin. All premiers were derived from Tianyi Huiyuan Biotechnology (Beijing, China) and summarized in Additional file 1: Table S1.

### Statistical analysis

For all the above analyses, p-value less than 0.05 was regarded as statistically significant. To compare the OS of patients between subgroups, we employed the Kaplan–Meier method with a two-sided log-rank test. When comparing the immune cell infiltration and immune pathway activation between the two groups, the Mann–Whitney test was used. All statistical analyses were accomplished with R software.

## Results

### Differential expression levels of m6A RNA regulators in normal and tumor samples in CRC

**Fig. 1:**
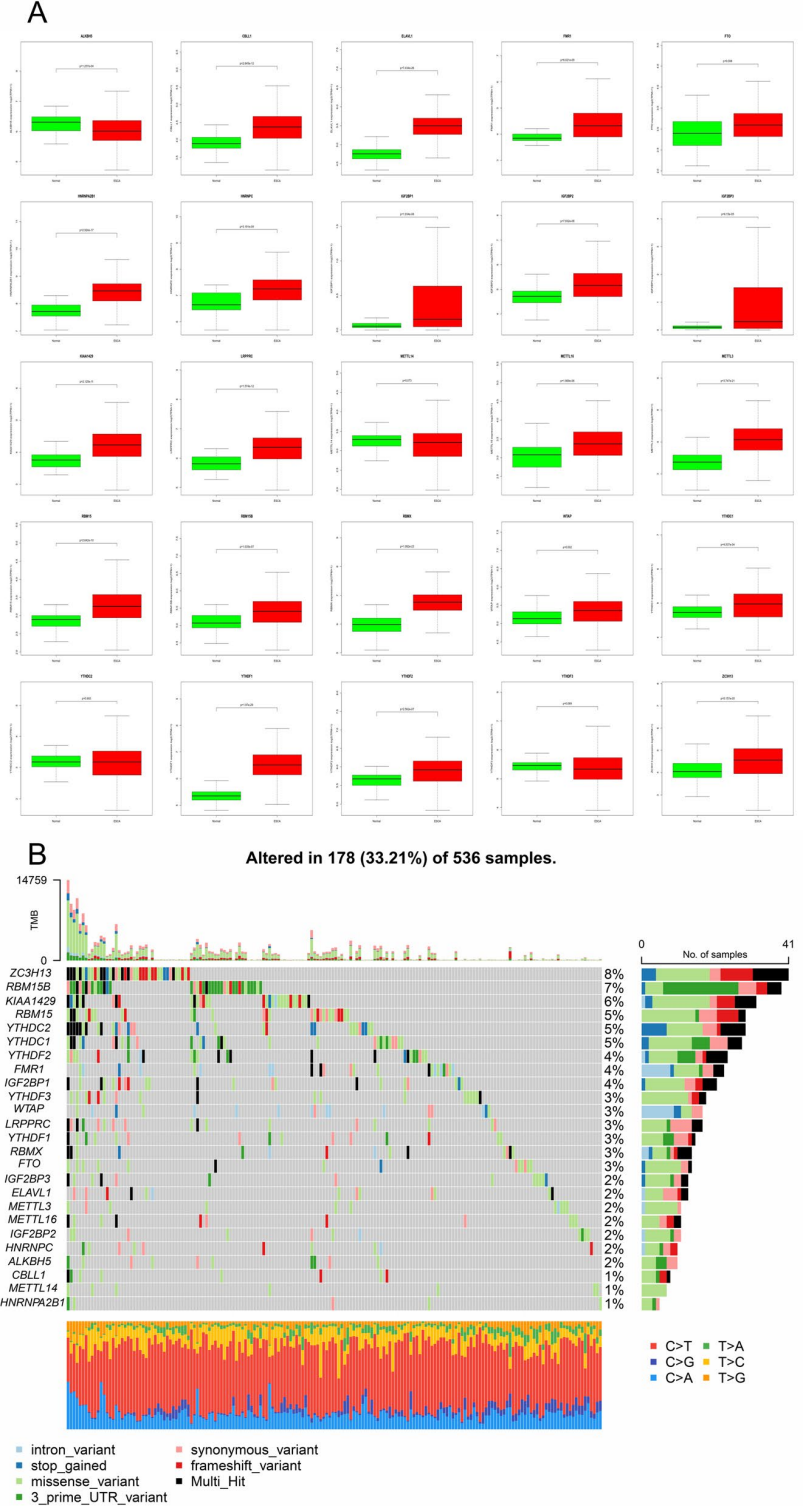
Differential expression patterns and mutation analysis of m6A regulators in CRC. **A** All m6A related genes expression were significantly different between tumor and control group except METTL14, YTHDF2 and YTHDF3. **B** 33.21%CRC patients contained mutated m6A related genes. C > T and T > G are the two main mutation types. The rainfall map shows high mutation genomic regions according to different SNP mutation types

The differential expression level of m6A related gene between cancer group and control group were analyzed. As shown in Fig. 1A, except METTL14, YTHDF2 and YTHDF3, all m6A related genes expression were significantly different between tumor and control group. This suggests that these m6A-related genes play important role in malignant progression of CRC.

### Mutation analysis of m6A regulators in CRC

To determine somatic mutations in CRC patients, we analyzed the mutation data using the R software package "Maftools". Next, the genetic alterations of m6A related genes in CRC patients were analyzed using the cBioPortal database as shown in Fig. 1B. In terms of mutation data, 178 of 536 CRC patients contained mutated m6A related genes. M6A related genes including ZC3H13 (8%), RBM15 (7%), KIAA1429 (6%), RBM15, YTHDC1, YTHDC2 are 4%, YTHDF2, WTAP, LRPPRC, YTHDF1, RBMX, FTO are 3%, METTL14, HNRNPA2B1 are 1% that are mutated. C > T and T > G are the two main mutation types, and the proportion of each sample is also shown in the histogram. The rainfall map shows high mutation genomic regions according to different SNP mutation types.

### M6A-related genes are enriched in regulation of mRNA metabolism process and spliceosome pathway

**Fig. 2:**
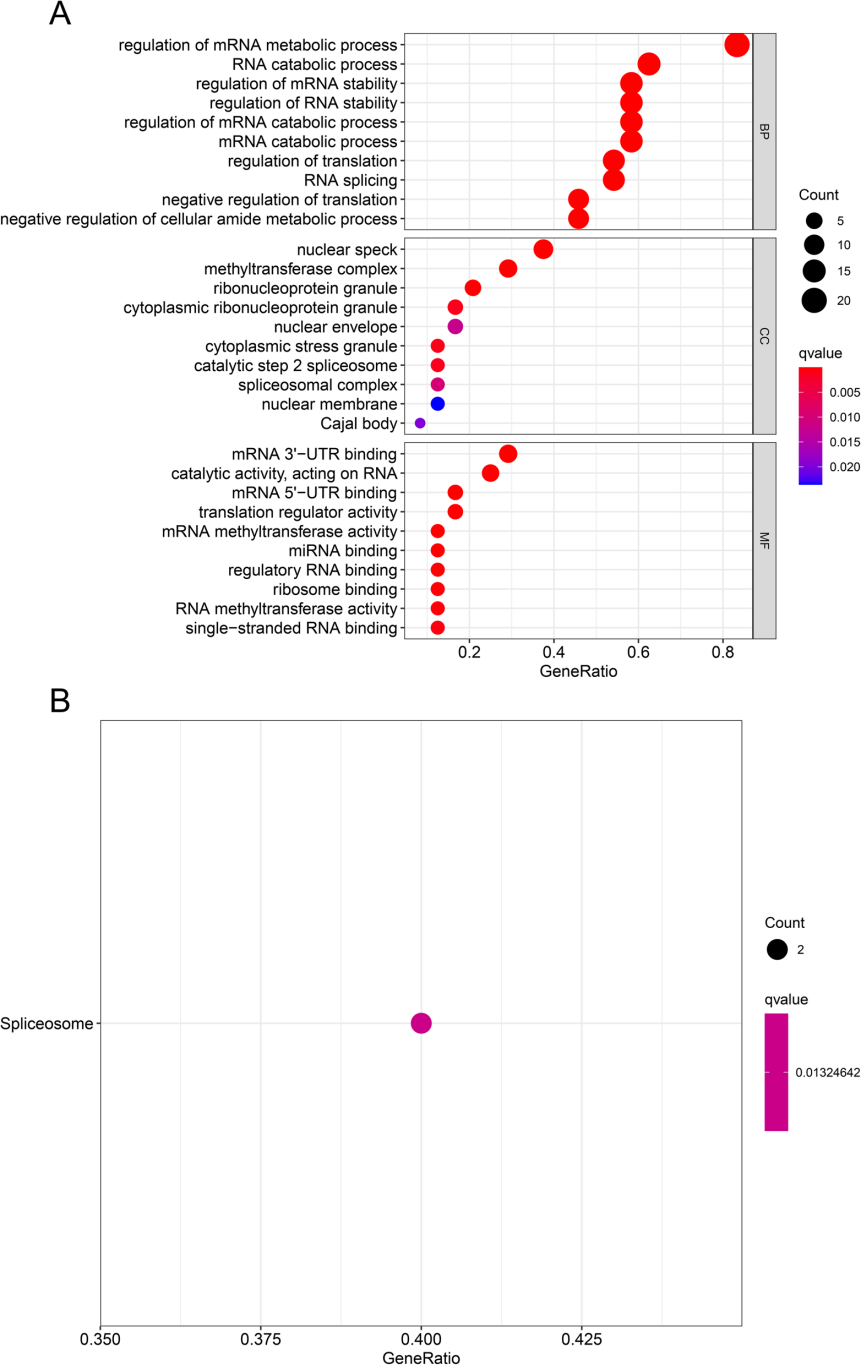
Go and KEGG enrichment analysis of m6a regulators in CRC. **A** Bar chart, **B** Bubble chart: mRNA metabolism process and spliceosome pathway enriched in GO and KEGG pathway

Go and KEGG enrichment analysis of m6A-related genes were performed by R package clusterprofiler to determine the signaling pathways and biological functions involved in CRC. It can be seen that mRNA metabolism process and spliceosome pathway enriched in GO and KEGG pathway (Fig. 2).

### Survival analysis of m6A gene in CRC

**Fig. 3:**
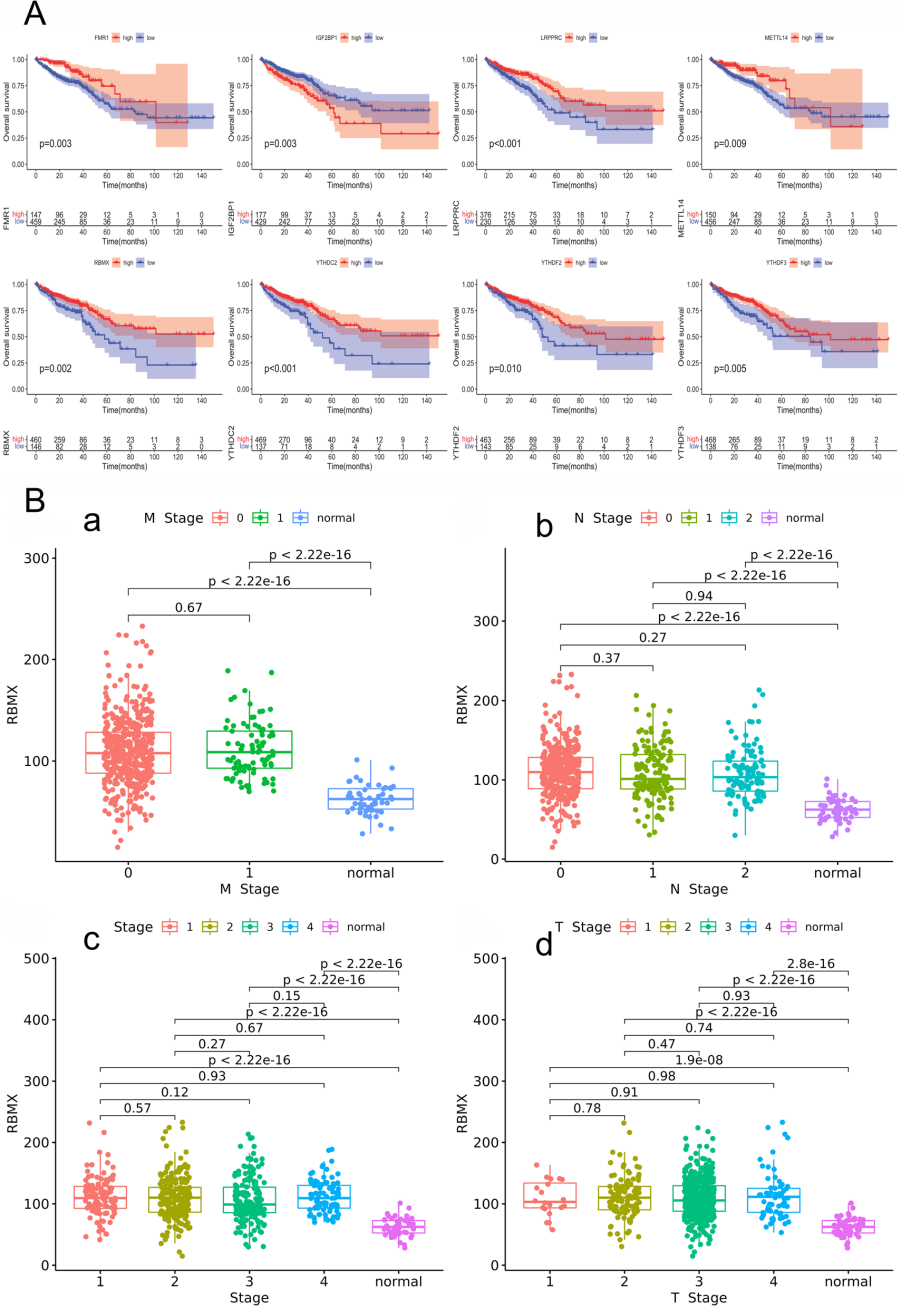
Survival analysis of m6A regulators in CRC. **A** Results of m6A regulators related with CRC prognosis were identified by Kaplan Meier analysis. **B** The correlation between expression of RBMX and clinical parameters, including stage, T, N, M in CRC

To further evaluate the prognostic value of m6A related genes in CRC, the relationship between their expression levels and overall survival in TCGA database was determined by Kaplan–Meier analysis. As shown in Fig. 3A, we identified 8 regulators related with CRC prognosis. Patients with high expressions of FMR1, LRPPRC, METTL14, RBMX, YTHDC2, YTHDF2, YTHDF3 and low expression of IGF2BP1 possess poor prognosis in CRC.

### Analysis of the correlation between m6A-related genes and clinical stage.

By comparing the expression of m6A related gene in the tumor group and the normal group (METTL14, YTHDF2 and YTHDF3 could not be excluded with statistical significance), mutation rates of m6A gene were all greater than 0, which could not be excluded. Prognostic value of m6A related gene in CRC (FMR1, LRPPRC, METTL14, RBMX, YTHDC2, YTHDF2, YTHDF3 prognostic related genes), Finally, five m6A related genes, RBMX, FMR1, LRPPRC, YTHDC2 and IGF2BP1, were screened out for subsequent analysis. We evaluated the relationship between expression patterns of RBMX, FMR1, LRPPRC, YTHDC2, IGF2BP1 and clinical parameters, including stage, T (tumor infiltration), N (lymphatic metastasis), M (distant metastasis) in CRC. As shown in Fig. 3B, we compared the correlation between the expression of RBMX in clinical factors stage, T, N, M stage in normal group and four neoplasm stages. Results showed a statistical difference between the normal group and the four neoplasm stages, T stage, N stage and M stage (p < 0.05). As shown in Additional file 2, Additional file 3, Additional file 4, we found that there was significant difference between normal group and four neoplasm stages for FMR1, IGF2BP1 and LRPPRC (p < 0.05, respectively). In addition, it was also found that there was a statistical difference between normal and T, N, M stage (p < 0.05). Moreover, we calculated the correlation between YTHDC2 expression in clinical factors stage between normal group and four neoplasm stages, there was no difference between the normal group and the four stages, T stage, N stage and M stage (p > 0.05) (Additional file 5).

### Correlation analysis between m6A related gene expression and immune infiltration in CRC

**Fig. 4:**
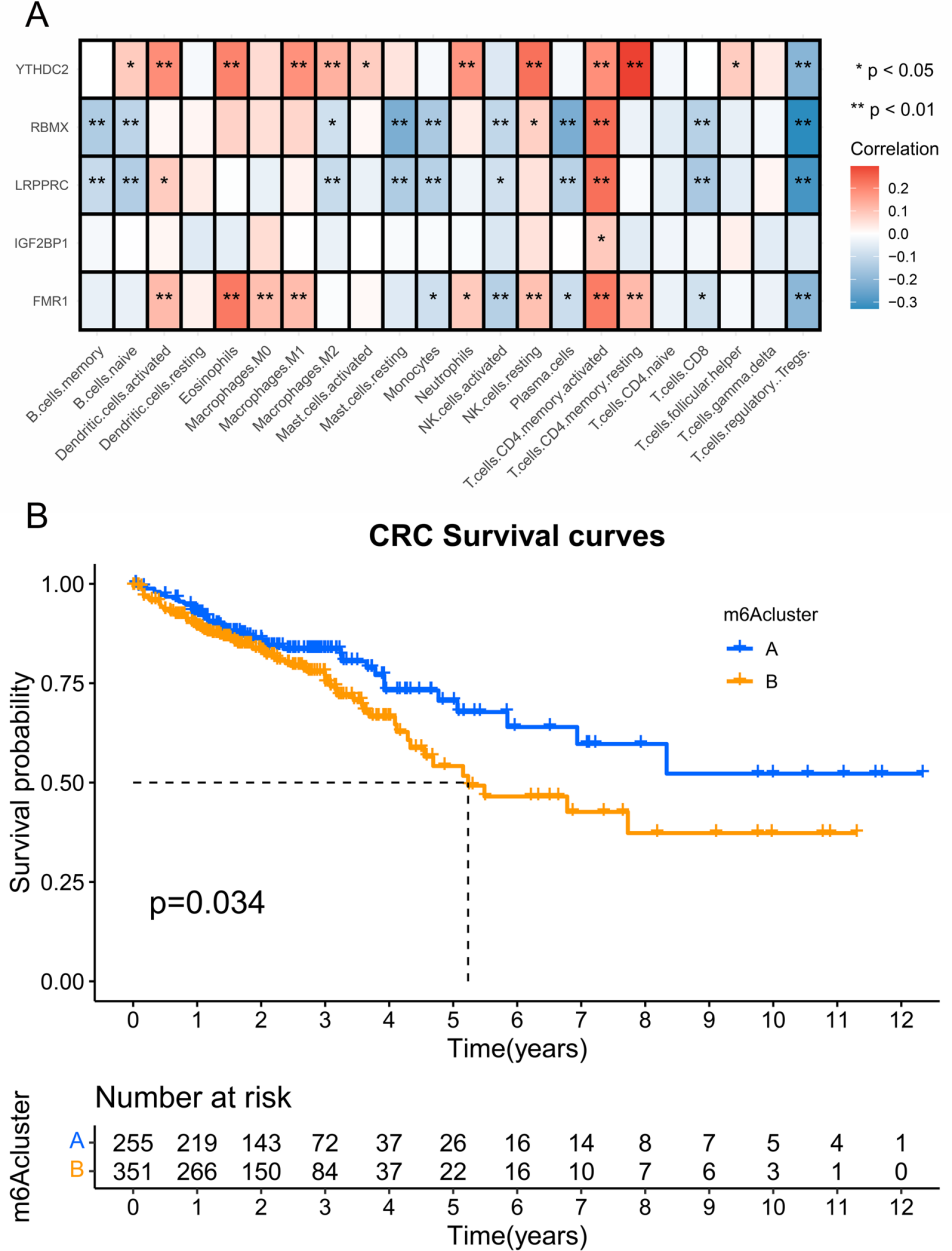
Correlation between m6A gene expression and immune infiltration, and clustering analysis in CRC. **A** FMR1, LRPPRC, RBMX, YTHDC2, HNRNPC and IGF2BP1 expression levels correlated with immune infiltration identified by CIBERSORTx database. **B** Results of significant differences of Kaplan–Meier curve between cluster A and Cluster B identified by R software package of ConsensusClusterPlus

Based on the CIBERSORTx database, we detected the correlation between m6A related gene and immune cell infiltration level in CRC. As shown in Fig. 4A, FMR1, LRPPRC, RBMX, YTHDC2 and HNRNPC expression significantly correlated with immune cell, IGF2BP1 expression only correlated with T.cells.CD4.memory.resting cell (p < 0.05).

### Correlation between m6A related gene and expression of immunomodulatory factors in CRC

In order to further explore the effect of m6A regulatory factor on tumor immune response, the correlation between m6A regulatory factor and immune regulatory factor expression was calculated. As shown in Additional file 6, Additional file 7, Additional file 8, the results showed that FMR1, IGF2BP1, LRPPRC, RBMX was negatively correlated with immunosuppressants, YTHDC2 was positively correlated with immunosuppressants. IGF2BP1, LRPPRC, RBMX was negatively correlated with immunostimulators, FMR1 and YTHDC2 was positively correlated with immunostimulators. FMR1, IGF2BP1, LRPPRC, RBMX was negatively correlated with MHC molecules, YTHDC2 positively correlated with MHC molecule.

### The m6 A regulator clusters CRC into two categories

**Fig. 5:**
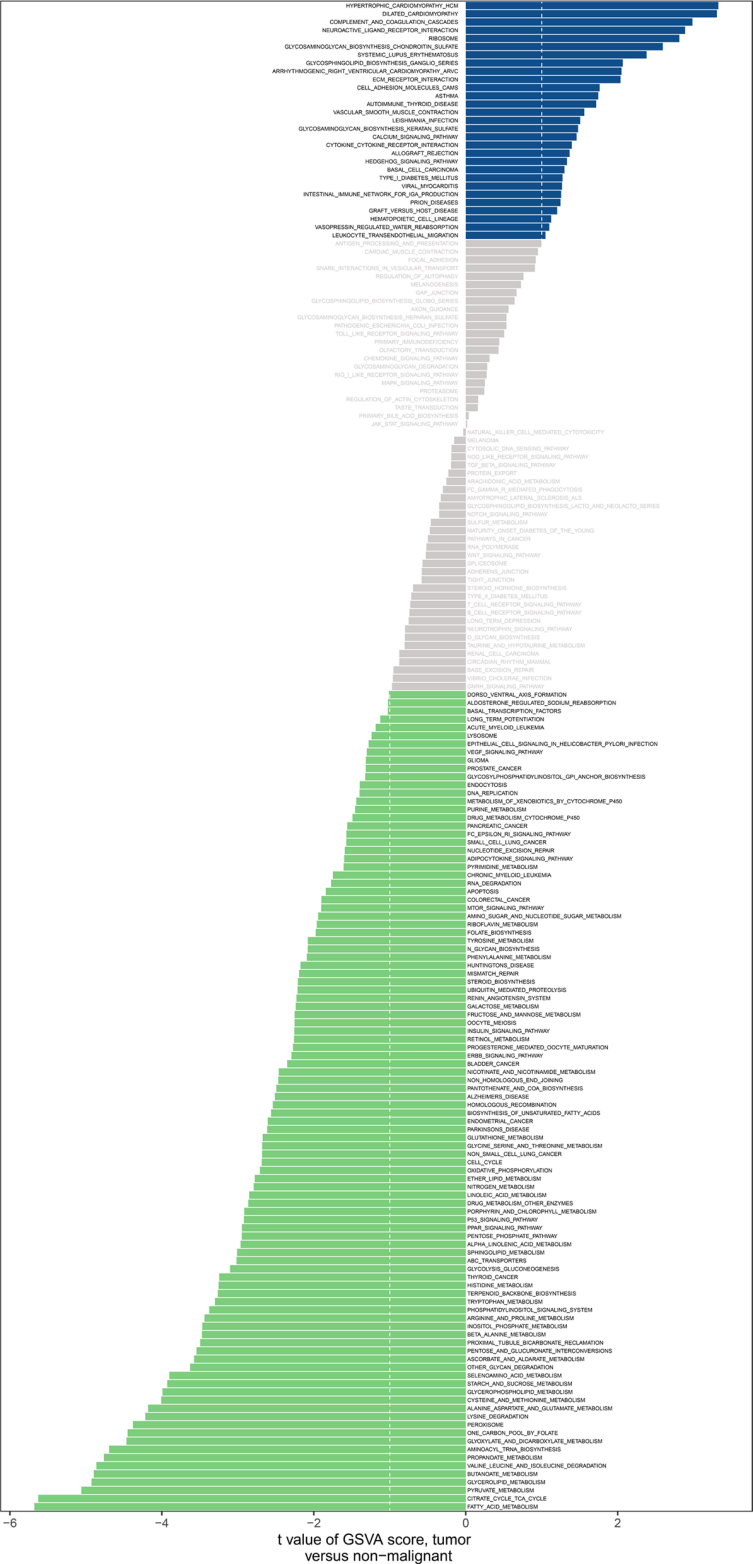
GSVA analysis between two different clusters. Results of GSVA analysis indicated that hypertrophic_cardiomyopathy_hcm and fatty_acid_metabolism were the most significant differences between the two clusters

Analysis of 638 patients based on TCGGA-COAD and TCGA-READ, we used the R software package of ConsensusClusterPlus to classify patients based on the expressions of FMR1, IGF2BP1, LRPPRC, RBMX and YTHDC2. We found that k = 2 clustering worked best. Two different modification patterns were finally determined using unsupervised clustering, including 304 cases of mode A and 334 cases of mode B, which we call cluster A and B, respectively. There are significant differences of Kaplan–Meier curve between cluster A and Cluster B (Fig. 4B). GSVA analysis showed that hypertrophic_cardiomyopathy_hcm and fatty_acid_metabolism were the most significant differences between the two clusters (Fig. 5).

### There were obvious differences in immune infiltration between the two clusters

**Fig. 6:**
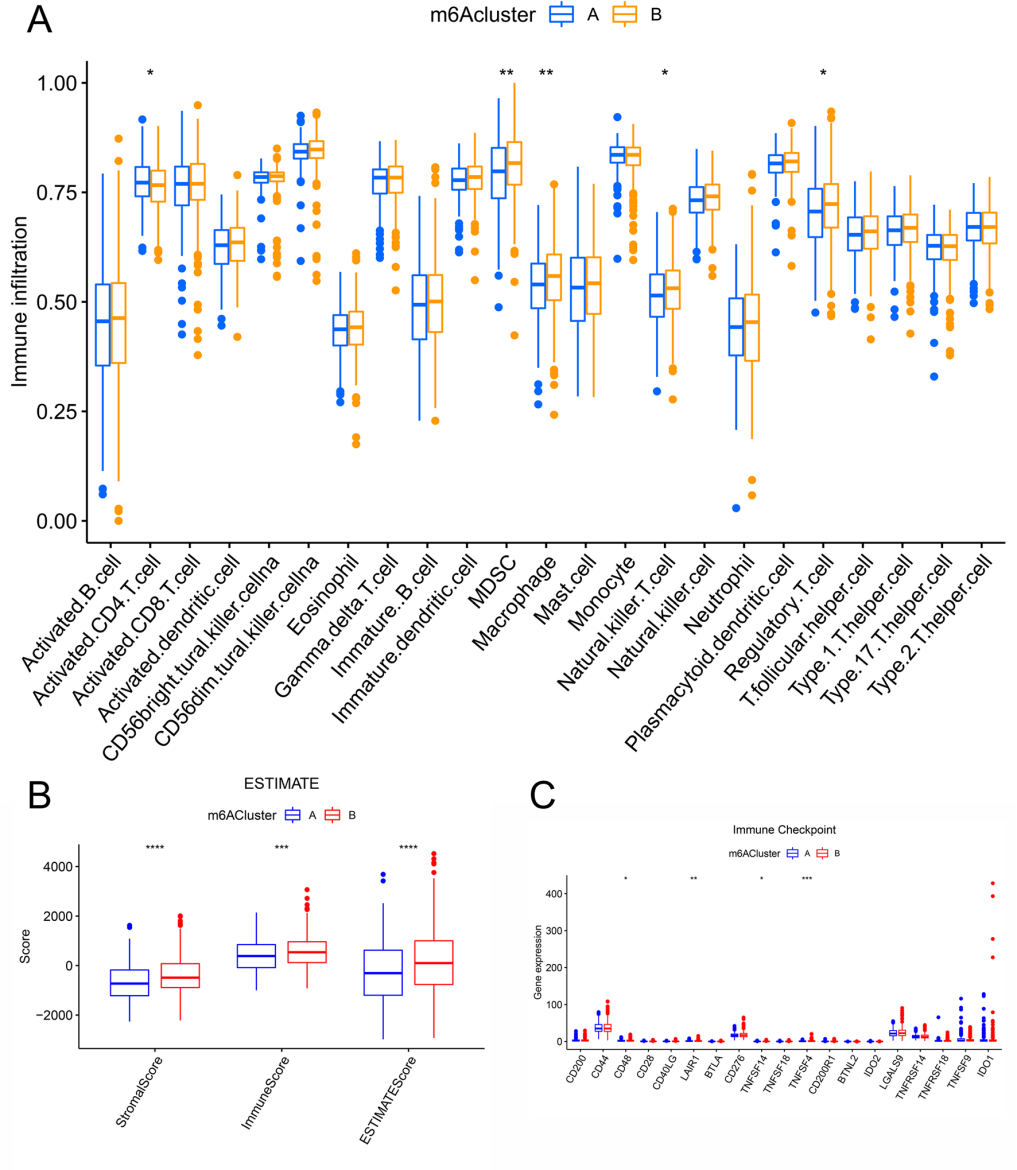
Analysis of immune infiltration between two different clusters. **A** Results of immune cell infiltration between the two clusters identified by ssGSEA. **B** Results of Immunescore, Stromalscore and Estimatescore between the two clusters by using estimate package. **C** CD44, CD40LG, CD276 and TNFSF18 were significantly difference between the two clusters identified by immune checkpoint analysis

We used ssGSEA to analyze the differences in immune cell infiltration between the two clusters. As shown in Fig. 6A, results indicated that Activated.CD4.T.cellna, MDSCna, Macrophagena, Natural.killer.T.cellna and Regulatory.T.cellna possess statistical difference between cluster A and cluster B. Immunescore and Stromalscore, Immunescore and Estimatescore were significantly different between the two clusters by using estimate package (Fig. 6B). Immune checkpoint analysis showed that CD44, CD40LG, CD276 and TNFSF18 were significant difference between the two clusters as shown in Fig. 6C. These results suggested that FMR1, LRPPRC, RBMX, YTHDC2 and IGF2BP1 might be involved in the development of CRC by participating in the immune regulation process of CRC.

#### GSEA analysis and tumor stem index analysis between Cluster A and Cluster B.

**Fig. 7:**
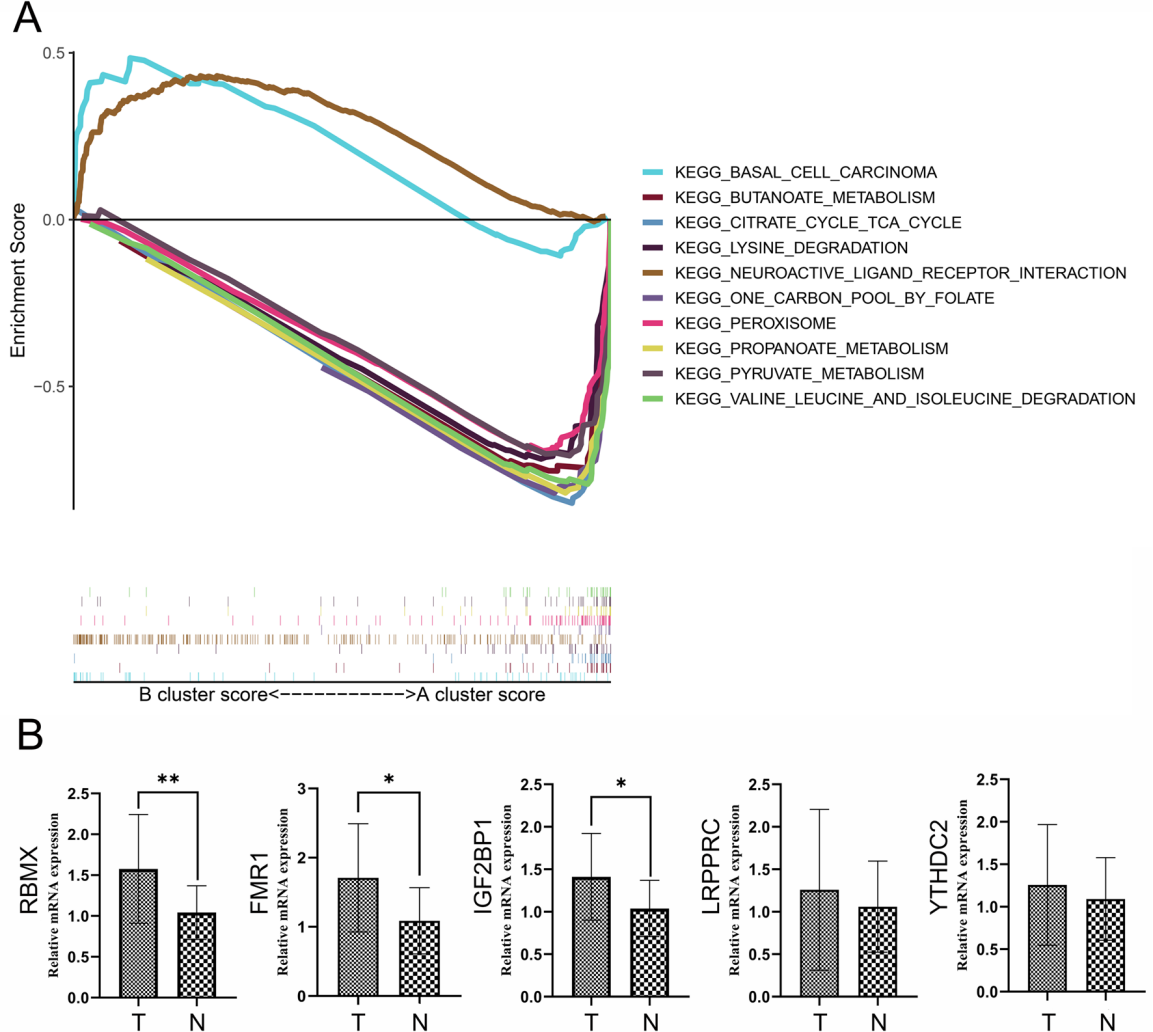
GSEA analysis between different clusters and validation of five potential biomarker for prognosis in CRC. **A** Results of enrichment pathways between the two clusters identified by GSEA analysis. **B** The expression patterns of RBMX, FMR1, IGF2BP1, LRPPRC and YTHDC2 were detected. RBMX expression was markedly elevated in cancerous tissues than in the normal colonic tissues

GSEA software was used to analyze the enrichment pathways between the two clusters. As shown in Fig. 7A, the enrichment pathways included basal cell carcinoma, Butancate metabolism and other pathways. Through OCLR algorithm, it can be seen that mRNAsi, EREG-MRNASI value has significant difference between the two clusters (p < 0.05) (Additional file 9), suggesting that FMR1, LRPPRC, RBMX, YTHDC2 and IGF2BP1 may affect CRC by regulating the pathway of basal cell carcinoma. These genes may also be involved in regulating the degree of tumor stemness.

#### Validation of five potential biomarker for prognosis in CRC

To clarify the role of RBMX, FMR1, IGF2BP1, LRPPRC and YTHDC2, we analyzed the mRNA expression patterns in human colorectal cancer specimens. The results indicated conspicuously higher RBMX expression in cancerous tissues (Fig. 7B).

## Discussion

Colorectal cancer is a major health challenge globally. Though there have been improvements of early detection and systemic treatment, some CRC patients still succumb to distant metastasis and recurrence due to limited treatment options [17, 18]. The majority of CRC are adenocarcinomas originating from colonic epithelial tissue which is the result of genetic mutations [6]. The extreme heterogeneity of CRC in histological subtyping, TNM staging and molecular subtypes brings certain differences in tumor biology, therapeutic effect and prognosis. There are four major types of CRC therapy according to different stages: surgery, radiotherapy, chemotherapy and immunotherapy. Recently, immunotherapy strategy against CRC being tested in a number of human clinical trials [19]. Tumor heterogeneity and its interaction with immune microenvironment remains a formidable challenge for CRC immunotherapy [20].

N6-methyladenosine (m6A), one such RNA modification is the N6-methylation found on adenosine (m6A) and 2'-O-methyladenosine (m6Am) regulated at post-transcriptionally level. It imposes a series of influence for RNA metabolism, such as splicing, translation, export and decay [21]. The variety of functionality played by this modification illustrates why m6A regulation correlated with various human cancer. Regulators of m6A are associated with tumor proliferation, angiogenesis and migration among different cancer types including papillary thyroid cancer, hepatocellular carcinoma, gastric cancer and colorectal cancer [22–24]. For instance, m6A related regulator, YTH m6A RNA-binding protein 1 (YTHDF1), plays important role in gastric carcinogenesis by controlling translation of FZD7 [25]. Aberrant expression of YTHDF1 correlated with more aggressive tumor phenotype and poor clinical prognosis. Overexpression of WTAP related to m6A methylation promotes hepatocellular carcinoma progression via the HuR-ETS1-p21/p27 axis [26]. WTAP serves as a potential therapeutic target of hepatocellular carcinoma therapy. These studies indicated the occurrence of m6A, a well-known modification with new epigenetic functions, perform regulatory mechanism in tumorigenesis.

There has been considerable research in the past years on the relationship between m6A and CRC [27]. As far as we're concerned, the function of m6A related prognostic biomarkers and regulatory mechanism in colorectal cancer, by far, has not been fully elucidated. It is highly necessary to clarify the prognostic value of m6A related regulators and its immune infiltration, a benefit for personalized immunotherapy in CRC.

In this work, to identify effective prognostic biomarkers and study the malignant biological properties of CRC, gene expression profile and clinical information of 536 CRC sample from TCGA dataset were evaluated by a series of bioinformatics analyses. First, we identified 25 differential expression levels of m6A regulators in CRC patients, the majority of m6A regulators were significantly different between CRC and normal group. Obviously, these candidate molecule plays important role in the progression of CRC. Furthermore, we analyzed the somatic mutations in CRC patients from the cBioPortal database. In terms of mutation data, 178 of 536 CRC patients contained mutated m6A related genes. To some extent, it suggests a role of m6A methylated modification in colorectal cancer susceptibility. Several cases of m6A methylated modification in cancer have been reported in the literature, and it is clear that the variant rs8100241 located in ANKLE1 was significantly associated with susceptibility of BRCA1 mutation triple negative breast cancer [28]. Besides, the association between m6A regulators aberrations and survival time in CRC obtained from TCGA was determined to further evaluate the prognostic value. Cox regression identified 8 m6A regulators related with CRC prognosis and patients with high expressions of FMR1, LRPPRC, METTL14, RBMX, YTHDC2, YTHDF2, YTHDF contribute to the poor prognosis of CRC patients. Moreover, low expression of IGF2BP1 possess poor prognosis in CRC patients. After further screening, we evaluated the relationship between expression of FMR1, LRPPRC, RBMX, YTHDC2, IGF2BP1 and clinical parameters, including stage, T (tumor infiltrating), N (lymphatic metastasis) and M (distant metastasis) in CRC. Obviously, we found that there was significant difference between normal group and four stages for FMR1, IGF2BP1, LRPPRC and RBMX. Nevertheless, there was no significant correlation between YTHDC2 and clinical stage of CRC patients. These m6A regulators may closely related to CRC severity. We all know that biomarkers can also reveal changes in a biological pathway that relate to disease progression. To further clarify the role of above five m6A regulators, we analyzed the mRNA expression patterns in human CRC samples. The results showed conspicuously higher RBMX, FMR1 and IGF2BP1 expression in tumor tissues, especially RBMX. These candidates can be regarded as potential therapeutic targets in CRC. RBMX is a ubiquitously expressed nuclear RNA binding protein which plays vital role in binding and stabilizing many proteins [29]. Some reports showed RBMX implicated in viral infection and cancer [30, 31]. RBMX serves as therapeutic target for hepatocellular carcinoma, because it promotes hepatocellular carcinoma development and reduce sorafenib sensitivity by targeting BLACAT1 [32]. RBMX plays tumor suppressor role in bladder cancer [33]. Neither is it clear what roles of RBMX played in CRC, it certainly looks worthy of further investigation.

The novelty and importance of m6A modification regulators enables researchers to gain a deeper level of understanding of CRC mechanisms related to novel immunotherapy approaches. Our study showed the superiority for relativity of prognosis and immune infiltration in CRC patients. In this study, we explored the roles of five m6A regulators and immune cell infiltration level in CRC. Primarily, we found that RBMX, FMR1, LRPPRC, YTHDC2 and HNRNPC expression significantly associated with immune cell, IGF2BP1 expression only correlated with T cells, CD4, memory cell. Considering the adaptive immune response influences the behavior of human cancer, we figured the tumor-infiltrating immune cells may therefore be a valuable prognostic tool in the therapy of CRC. The majority of CRC patients are microsatellite-stable genetic subtype, a molecular indicator of proficient DNA mismatch repair. Compared with microsatellite-stable genetic subtype, high tumor mutational burden and neoantigen load in microsatellite instability tumors favors the infiltration of immune effector cells [34]. It is reported that RBMX and HNRNPC can predict the prognosis of head and neck squamous cell carcinoma and are related to immune infiltration [35]. Additionally, YTHDC2 was significantly associated with tumor immune infiltration in skin cutaneous melanoma and brain lower grade glioma [36]. we arrived at the same general conclusion that YTHDC2 was positively correlated with immune cells.

Immunotherapy is the most exciting and most promising development in cancer research [37]. M6A modification is heavily involved in modulation of immune responses, and how immune responses regulate m6A modification remains elusive in CRC. We hold opinion that immune cell infiltration of primary non-metastatic tumors is a strong prognostic factor for survival in CRC patients. Hence, we examined the correlation between m6A regulatory factor and immune regulatory factor. We found that FMR1, IGF2BP1, LRPPRC, RBMX was negatively correlated with immunosuppressants, YTHDC2 was positively correlated with immunosuppressants. IGF2BP1, LRPPRC, RBMX was negatively correlated with immunostimulators, FMR1 and YTHDC2 was positively correlated with immunostimulators. FMR1, IGF2BP1, LRPPRC, RBMX was negatively correlated with MHC molecules, YTHDC2 positively correlated with MHC molecule. Immune cells means a large fraction of the tumor microenvironment which plays crucial role in mediating pro-tumor and anti-tumor immune responses. There are some studies suggesting the same theoretical for immunomodulation in different cancer types [38, 39]. It follows that immunomodulation is closely linked with tumor relapse and prognosis in CRC. Collectively, the mechanism of above m6A related regulators with immune microenvironment has not been elucidated thoroughly now. Some of these regulators play important role in epigenetic modification and immune infiltration, which may serves as potential biomarker for prognosis in CRC patients.

## Conclusions

Our study efficiently constructed an m6A related regulators prognostic signature and evaluated the involvement of immune infiltration in colorectal cancer patients. The signature might provide five candidate targets (RBMX, FMR1, IGF2BP1, LRPPRC, YTHDC2) associated with specific clinical features, prognosis and improvement in immunotherapy for patients with colorectal cancer.

## Supplementary Information

**Additional file 1: Table S1.** Primers.

**Additional file 2.** FMR1: Analysis of the correlation between m6a regulators and clinical stage. Results of correlation between FMR1 and clinical stage (*p* > 0.05).

**Additional file 3.** IGF2BP1: Analysis of the correlation between m6a regulators and clinical stage. Results of correlation between IGF2BP1 and clinical stage (*p* > 0.05).

**Additional file 4.** LRPPRC: Analysis of the correlation between m6a regulators and clinical stage. Results of correlation between LRPPRC and clinical stage (*p* > 0.05).

**Additional file 5.** YTHDC: Analysis of the correlation between m6a regulators and clinical stage. Results of correlation between YTHDC2 and clinical stage (*p* > 0.05).

**Additional file 6.** Correlation between m6A regulators and immunomodulatory factors in CRC: FMR1, IGF2BP1, LRPPRC, RBMX was negatively correlated with immunosuppressants, YTHDC2 was positively correlated with immunosuppressant.

**Additional file 7.** Correlation between m6A regulators and immunomodulatory factors in CRC. IGF2BP1, LRPPRC, RBMX was negatively correlated with immunostimulators, FMR1 and YTHDC2 was positively correlated with immunostimulator.

**Additional file 8.** Correlation between m6A regulators and immunomodulatory factors in CRC: FMR1, IGF2BP1, LRPPRC, RBMX was negatively correlated with MHC molecules, YTHDC2 positively correlated with MHC molecule.

**Additional file 9.** Stem index analysis between different clusters. mRNAsi, EREG-MRNASI value had statistical difference between the two clusters (*p* < 0.05).

## Data Availability

The datasets used and analysed during the current study are available from TCGA database (http://portal.gdc.cancer.gov/), cBioPortal database (https://www.cbioportal.org/) and TISIDB database (http://cis.hku.hk/TISIDB/). The data generated and analyzed during this study are described in the following data record: https://doi.org/10.6084/m9.figshare.22492333.

## Abbreviations

CRC: Colorectal cancer
m6A: N6-methyladenosine
MSS: Microsatellite stable
MSI-L: Microsatellite instability-Low
MSI-H: Microsatellite instability-high
PD-1: Programmed death 1
TCGA: The Cancer Genome Atlas
