# Supplementary material for: Comprehensive analysis of m6A related gene mutation characteristics and prognosis in colorectal cancer
Source: BMC Med Genomics. 2023 May 16;16:105. doi: 10.1186/s12920-023-01509-8 (PMC10186803; doi:10.1186/s12920-023-01509-8)
Supplement: Supplementary file 1 — Additional file 1: Table S1. Primers. [file 12920_2023_1509_MOESM1_ESM.docx]

Table S1: Primers

| Gene symbol | Primer sequence | Length | Annealing temperature |
| --- | --- | --- | --- |
| ***FMR1*** | F:CGACGATCACTCCCGAACA  R:TGCTGACCATCCACGCTGT | 216 | 60 |
| ***IGF2BP1*** | F:ACTTTGTAGGGCGTCTCATTGG  R:GGCAGCCACATCATTCTCAT | 215 | 60 |
| ***LRPPRC*** | F:GTGAGGGAGGGTTGATGGC  R:GCGGAGGTTGCAGTGAGCT | 299 | 60 |
| ***RBMX*** | F:ATTGGTGGGCTTAATACGGAA  R:TGGTTTGGTGGCTTGTTCC | 228 | 59 |
| ***YTHDC2*** | F:GATTCCCACGGTCTTTGTCAT  R:CTGCGATCTTCACCTCCTCAT | 224 | 59 |
