# Supplementary material for: Comprehensive analysis of m6A related gene mutation characteristics and prognosis in colorectal cancer
Source: BMC Med Genomics. 2023 May 16;16:105. doi: 10.1186/s12920-023-01509-8 (PMC10186803; doi:10.1186/s12920-023-01509-8)
Supplement: Supplementary file 4 — Additional file 4. LRPPRC: Analysis of the correlation between m6a regulators and clinical stage. Results of correlation between LRPPRC and clinical stage (p > 0.05). [file 12920_2023_1509_MOESM4_ESM.pdf]

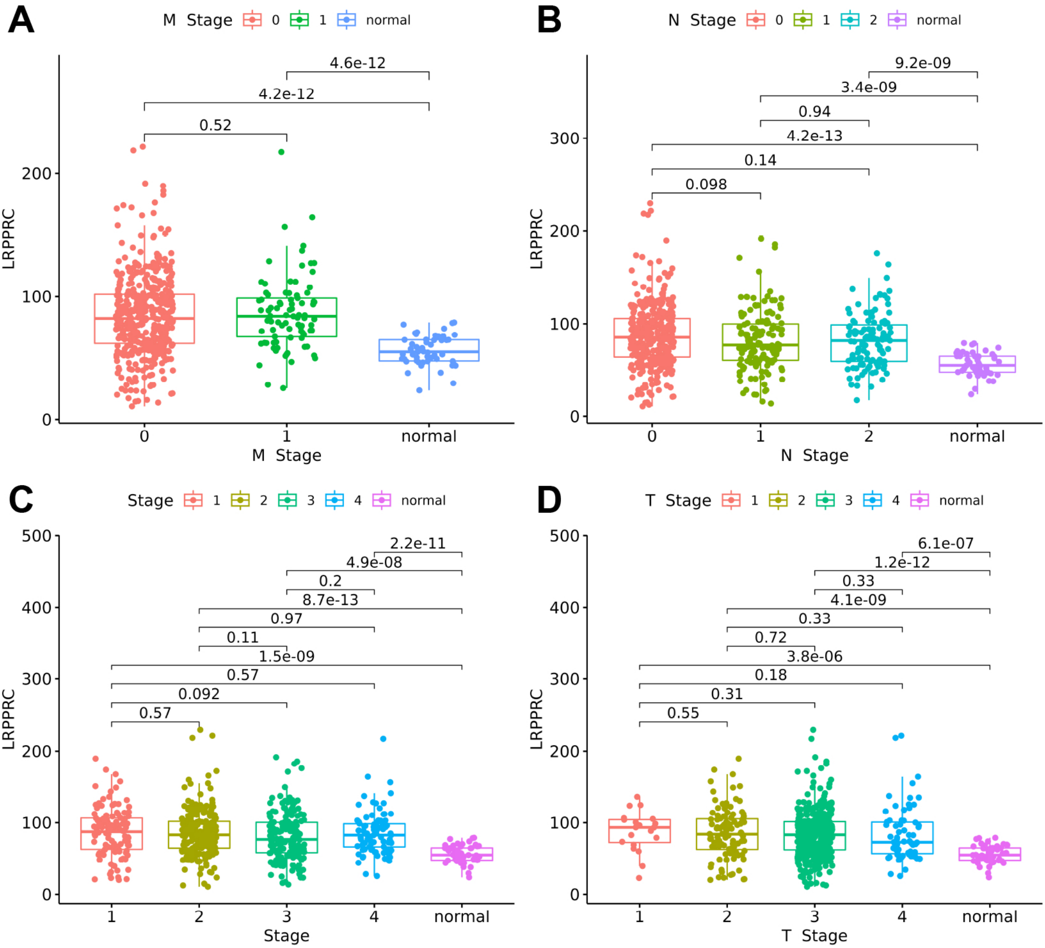

additional file 4-LRPPRC:
Analysis of the correlation between m6a regulators and clinical stage.
Results of correlation between LRPPRC and clinical stage($p<0.05$).
