# Supplementary material for: Comprehensive analysis of m6A related gene mutation characteristics and prognosis in colorectal cancer
Source: BMC Med Genomics. 2023 May 16;16:105. doi: 10.1186/s12920-023-01509-8 (PMC10186803; doi:10.1186/s12920-023-01509-8)
Supplement: Supplementary file 8 — Additional file 8. Correlation between m6A regulators and immunomodulatory factors in CRC: FMR1, IGF2BP1, LRPPRC, RBMX was negatively correlated with MHC molecules, YTHDC2 positively correlated with MHC molecule. [file 12920_2023_1509_MOESM8_ESM.pdf]

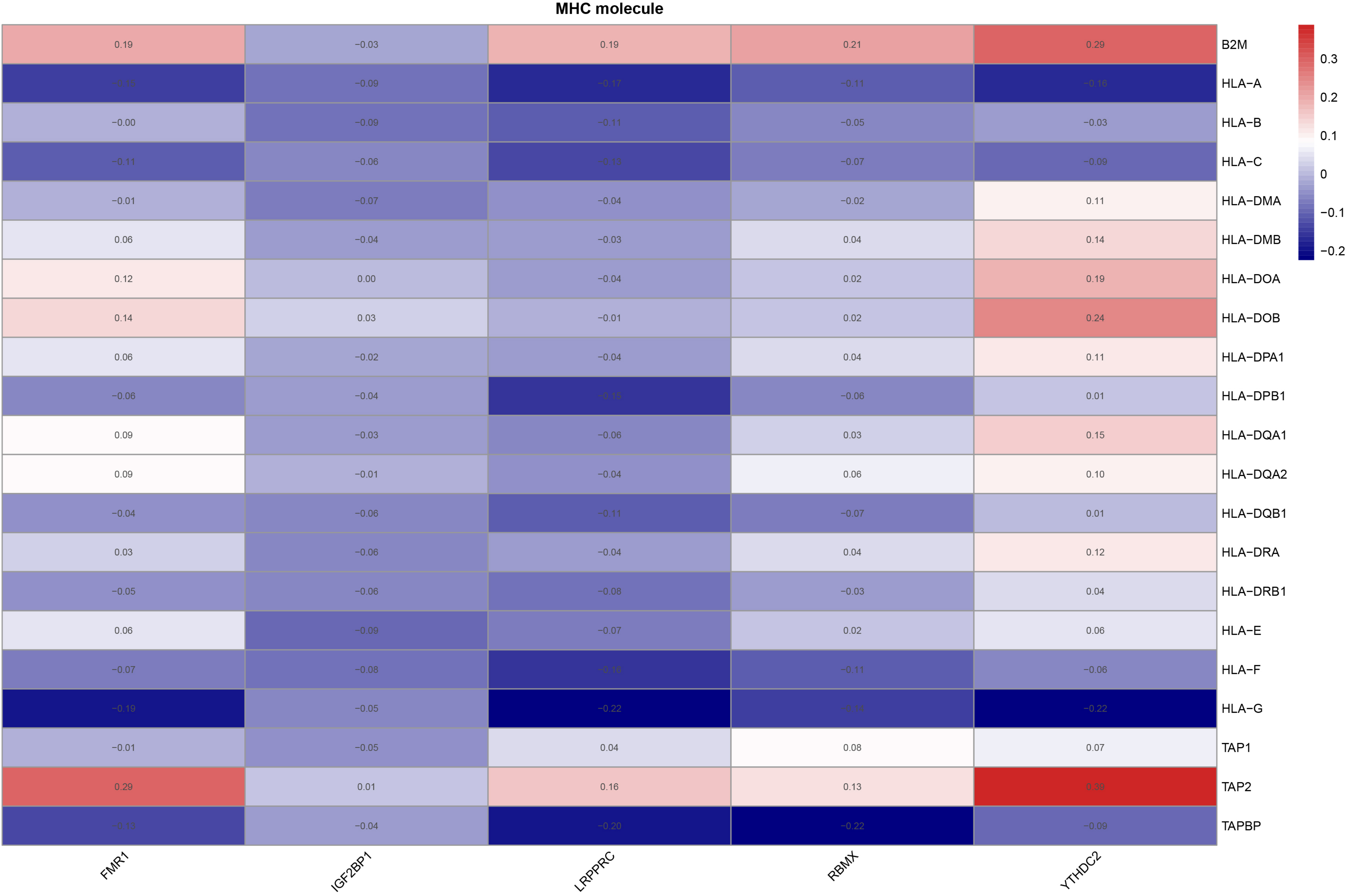

additional file 8 -Correlation between m6A regulators and immunomodulatory factors in CRC:
FMR1, IGF2BP1, LRPPRC, RBMX was negatively correlated with MHC molecules, YTHDC2 positively correlated with MHC molecule.
