# Supplementary material for: Comprehensive analysis of m6A related gene mutation characteristics and prognosis in colorectal cancer
Source: BMC Med Genomics. 2023 May 16;16:105. doi: 10.1186/s12920-023-01509-8 (PMC10186803; doi:10.1186/s12920-023-01509-8)
Supplement: Supplementary file 9 — Additional file 9. Stem index analysis between different clusters. mRNAsi, EREG-MRNASI value had statistical difference between the two clusters (p < 0.05). [file 12920_2023_1509_MOESM9_ESM.pdf]

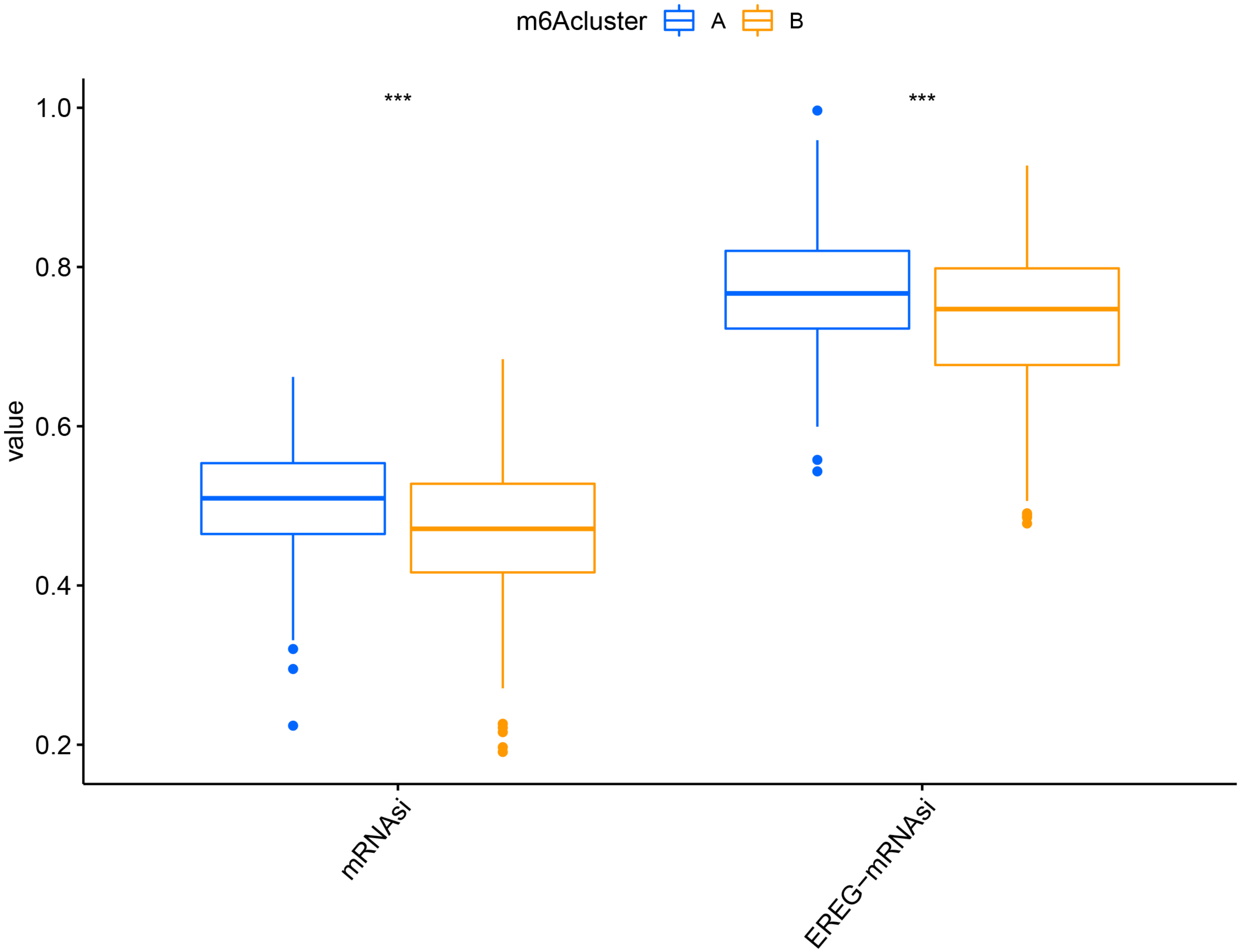

additional file 9 -Stem index analysis between different clusters. mRNAsi, EREG-MRNASI value had statistical difference between the two clusters (p<0.05).
